# Primary clear cell adenocarcinoma of the seminal vesicle with ovarian homology: A rare case report

**DOI:** 10.1097/MD.0000000000039020

**Published:** 2024-11-08

**Authors:** Yang Yuan, Yunlong Li, Shihao Zhang, Dingli Hu, Song Xu, Tingting Gu

**Affiliations:** a Department of Urology, Kunshan First People’s Hospital, Affiliated to Jiangsu University, Kunshan, Jiangsu, China; b Department of Pathology, Kunshan First People’s Hospital, Affiliated to Jiangsu University, Kunshan, Jiangsu, China.

**Keywords:** clear cell adenocarcinoma, pathology, primary, seminal vesicle cancer

## Abstract

**Rationale:**

Primary seminal vesicle adenocarcinoma is a rare type of male reproductive system tumor, primarily manifesting as papillary adenocarcinoma. Meanwhile, clear cell adenocarcinoma (CCA) is a common malignancy in the female reproductive system. Therefore, the occurrence of CCA in the seminal vesicle, showing ovarian homology, is even rarer. This pathological type of seminal vesicle cancer has been seldom reported.

**Patient concerns:**

A 43-year-old male patient presented to the outpatient clinic with a decade-long history of intermittent hemospermia accompanied by a 2-month sensation of dragging in the left lower abdomen. Following an MRI scan that indicated a seminal vesicle mass, he was admitted for further treatment.

**Diagnosis:**

The MRI revealed 2 abnormal signal mass lesions located between the left side of the pelvic cavity, the prostate, and the left seminal vesicle, primarily exhibiting long T1 and long T2 cystic characteristics. The CT scan with enhancement showed mixed density mass shadows in the left seminal vesicle area of the pelvic cavity, with mild arterial phase enhancement and continued enhancement in the portal phase. Physical examination revealed mild tenderness in the lower left abdomen. Digital rectal examination detected a hard mass above the left prostate, with no bloodstain on the glove, and no other significant abnormalities were observed. Routine urinalysis and biochemistry did not reveal any notable abnormalities. Tumor markers were all within normal ranges.

**Interventions:**

The tumor was completely excised laparoscopically and sent for pathological examination. Nine days postoperatively, the patient was successfully discharged.

**Outcomes:**

Postoperative pathology indicated primary CCA of the seminal vesicle. During a 20-month follow-up via telephone, the patient reported a generally good condition without significant discomfort.

**Lessons:**

CCA occurring in the seminal vesicle is extremely rare, and radical surgical excision is the primary treatment method.

## 1. Introduction

Clear cell adenocarcinoma (CCA) is believed to originate from epithelial tissues derived from the Müllerian duct. In females, this duct develops into the uterus, fallopian tubes, and a portion of the vagina, whereas it typically regresses in males. We report a case involving a 43-year-old male who presented with intermittent hematospermia for over a decade, accompanied by a sensation of heaviness in the left lower abdomen for 2 months. Examination revealed a seminal vesicle mass, which was surgically removed. Postoperative pathology indicated that the histological morphology and immunophenotype of the tumor were entirely consistent with ovarian CCA, leading to a diagnosis of seminal vesicle CCA with ovarian homology. The discovery of this pathological type of seminal vesicle cancer provides insights into the developmental mechanisms of the male and female reproductive systems.

## 2. Case report

**Figure 1.**
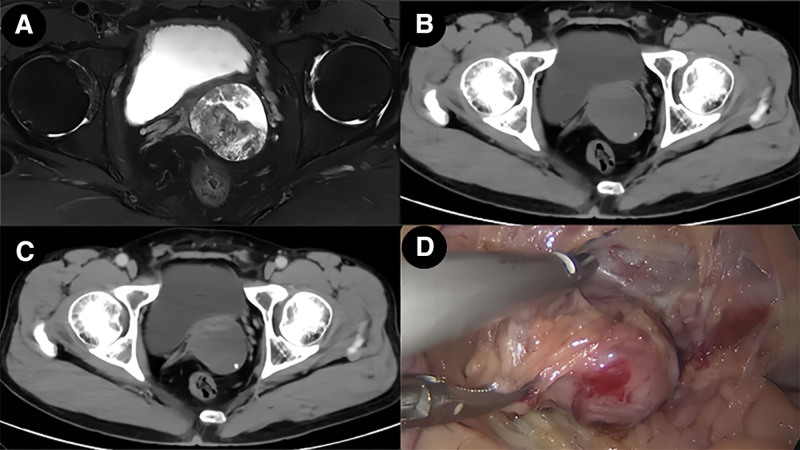
Patient’s pelvic MRI and CT plain scan + enhancement, intraoperative pictures. (A) MRI shows 2 mass-like abnormal signal foci between the left side of the pelvis, prostate, and left seminal vesicle, mostly presenting as long T1 and long T2 cystic. (B, C) CT plain scan + enhancement shows a mixed density mass in the left seminal vesicle area of the pelvis, with mild enhancement in the arterial phase and continued enhancement in the portal phase. (D) Intraoperatively, a round tumor about 5 cm in size was observed.

**Figure 2.**
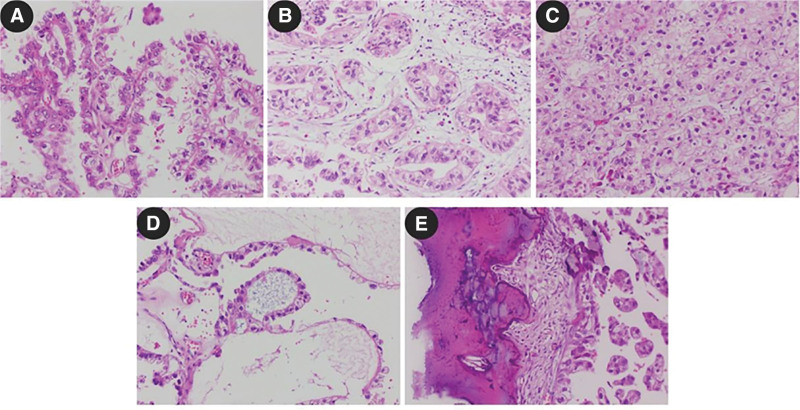
Seminal vesicle clear cell adenocarcinoma histological morphology, H&E staining, IHC × 200.

**Figure 3.**
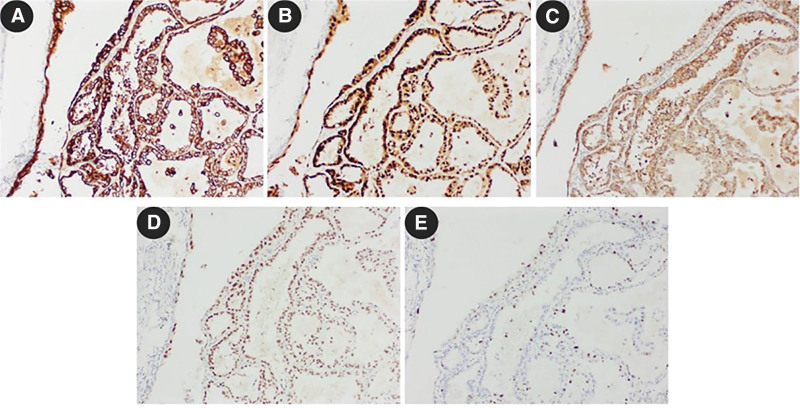
Immunohistochemical staining results of seminal vesicle clear cell adenocarcinoma (EnVision method, IHC × 200). (A) Cancer cells CK7 (+). (B) Cancer cells PAX-8 (+). (C) Cancer cells Napsin A (+). (D) Cancer cells HNF-1β (+). (E) Cancer cells Ki67 (+ about 15%).

**Figure 4.**
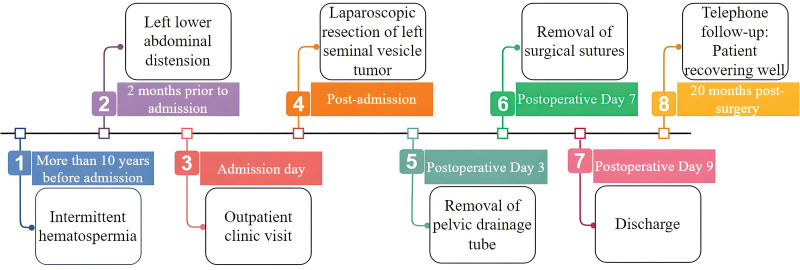
Timeline of patient presentation, treatment, and recovery.

Patient, a 43-year-old male, was admitted to the Urology Department with a history of intermittent hemospermia for over ten years and a sensation of dragging in the left lower abdomen for 2 months. The patient did not report urgency, frequency, dysuria, difficulty in urination, or significant recent weight changes. Outpatient pelvic MRI suggested: 2 abnormal signal masses between the left pelvic cavity, prostate, and left seminal vesicle, poorly demarcated from the prostate and left seminal vesicle, measuring 48 × 46 × 51 mm and 21 × 33 × 18 mm (anteroposterior × transverse × craniocaudal), mostly showing long T1 and long T2 cystic signals, with scattered flaky mixed short T1 and T2 signals inside, mild diffusion restriction in DWI, and partial connection between the 2 lesions. Multiple isointense T1 and short T2 fluid levels were observed in the left seminal vesicle. The right seminal vesicle showed high signal on T1WI and iso-high signal on T2WI (Fig. 1A). Pre-hospital CT scan with enhancement: A 45 × 58 mm mixed density mass shadow was seen in the left seminal vesicle area of the pelvic cavity, with dot-like calcifications, mild arterial phase enhancement, and continuous portal phase enhancement (Fig. 1B and C). Physical examination: Mild tenderness in the lower left abdomen, hard mass palpable above the left side of the prostate during digital rectal examination, glove unstained with blood, and no other significant abnormalities. Routine urinalysis and biochemistry showed no significant abnormalities. Tumor markers were within normal ranges. After completing preoperative examinations and excluding contraindications for surgery, a laparoscopic left seminal vesicle tumor excision was performed under general anesthesia. Intraoperatively, a 5.0 cm spherical mass was observed in the left seminal vesicle, with adhesions to surrounding tissues (Fig. 1D). The tumor was separated with an ultrasonic scalpel, transected at the base of the prostate, and the seminal vesicle tumor was completely excised. Postoperatively, the patient’s vital signs and drainage were closely monitored. Treatment included urinary catheterization, prophylactic antibiotics, hemostasis management, fluid resuscitation, and pain control. Dietary guidance was provided to the patient and their family, along with education on urinary catheter care and assistance with turning. The pelvic drain was removed on postoperative day 3, the incision sutures were removed on day 7, and the patient was discharged on day 9 with a satisfactory recovery. Postoperative pathology: Microscopically, tumor cells were mostly single-layered cuboidal epithelium, with distinct cell membranes, clear cytoplasm, and parts of lightly eosinophilic cytoplasm. The nuclei were slightly round, varying in size, often irregular in shape, with distinct nuclear membranes, fine granular chromatin, and moderately sized prominent eosinophilic nucleoli. Tumor cells were arranged in glandular, papillary structures, and nail-like patterns, with some solid areas; these structures often coexisted (Fig. 2A–C). Pink secretions and degenerated tumor cells forming mucus were commonly seen in glandular lumens (Fig. 2D). Local areas of hemorrhagic degeneration and necrosis, and interstitial ossification were observed in the tumor (Fig. 2E). Immunohistochemistry results: PLAP (−), AFP (−), α-inhibin (−), CD117 (−), AE1/AE3 (+), NapsinA (+), PSA (−), RCC (−), CK8/18 (+), Glypican-3 (partially +), Calretinin (−), CD30 (−), SALL-4 (−), Ki67 (about 15% +), PAP (−), CK7 (+), CEA (−), CDX-2 (−), CA125 (−), PAX-8 (+) (Fig. 3). Combined with HE morphology and immunohistochemical phenotype, it was consistent with primary CCA of the seminal vesicle. No cancerous tissues were found in the vas deferens and surgical margins. A telephone follow-up was conducted 20 months after the surgery. The patient reported a good general condition without significant discomfort or recurrence of hematospermia and expressed high satisfaction with the treatment outcome (Fig. 4).

## 3. Discussion

Primary seminal vesicle adenocarcinoma (PSVA) is a rare malignancy of the male reproductive system with limited epidemiological data, with primary cases being less common than secondary. It presents a wide age range, from 19 to 90 years, predominantly affecting individuals over 50.^[1]^ The main clinical manifestations include lower abdominal or perineal pain, dysuria, and hematospermia. Early diagnosis of PSVA is challenging due to symptom overlap with more common diseases and the absence of distinct laboratory abnormalities. Physical examination primarily involves rectal palpation for tenderness and masses, though differentiation from the prostate is difficult. In the early stages of the disease, CT, MRI, and ultrasound are beneficial for tumor localization, typically revealing solid or cystic masses between the rectum and either the prostate or bladder.^[2–5]^ While imaging assists in determining the origin and location of seminal vesicle carcinoma, histopathological examination remains the gold standard for diagnosis. Dalgaard et al, in 1956, established the first diagnostic criteria for PSVA: histologically confirmed cancer predominantly confined to the seminal vesicles, exclusion of concurrent primary cancers elsewhere, and the tumor should resemble the non-neoplastic structure of the seminal vesicles.^[6]^ Thus, ruling out primary malignancies in adjacent organs like the prostate, bladder, and colorectum is crucial, as seminal vesicle carcinoma often presents at an advanced stage and is susceptible to invasion from these neighboring malignancies. In this case, the patient experienced intermittent hematospermia for over ten years without formal diagnosis or treatment and presented with left lower abdominal discomfort, lacking distinctive diagnostic features.

PSVA originates from the epithelial cells of the seminal vesicle glands, with a minority presenting as bilateral tumors.^[4,7]^ It primarily manifests as papillary adenocarcinoma, but other reported pathological types include squamous carcinoma, neuroendocrine tumors, Burkitt lymphoma, and epithelial-stromal tumors. CCA is a rare tumor of the urogenital tract, predominantly occurring in females with rare reports in males. Through literature search, it was found that in 1967, Gaur et al reported the first case of primary CCA of the seminal vesicle. Currently, only 4 cases with complete clinical data have been reported, among which 2 cases presented with hemospermia, 1 case presented with a testicular mass, and 1 case presented with intermittent painless hematuria.^[7–10]^ This case highlights the diverse clinical presentations of seminal vesicle cancer, with the patient presenting with left lower abdominal distension. This variability likely stems from differences in tumor size and location leading to varying compressive symptoms, as well as the inherent aggressiveness of the tumor. In this case, the postoperative pathology indicated primary CCA of the seminal vesicle. Immunohistochemical staining was positive for AE1/AE3, NapsinA, CK8/18, CK7, PAX-8, and partially positive for Glypican-3, with about 15% positivity for Ki67. It was negative for PLAP, AFP, α-inhibin, CD117, PSA, RCC, Calretinin, CD30, SALL-4, PAP, CEA, CDX-2, and CA125.Posenato and others consider the immunohistochemical markers CA125 (+), CK7 (+), CK20 (−), PSA, and PSAP (−) to be characteristic of primary seminal vesicle carcinoma, with PAX-8 expression confirming the tumor’s seminal vesicle origin.^[11]^ In this case, the clinical and immunohistochemical findings, excluding other primary tumors such as prostate, colorectal, renal cell, and bladder cancer, led to the diagnosis of primary seminal vesicle CCA.CA125 is a tumor marker expressed in Müllerian duct-derived tumors. The seminal vesicle gland, derived from the Wolffian duct, is a component of the male reproductive system. Most seminal vesicle carcinoma patients abnormally express CA125, indicating embryonic homology between male and female reproductive systems. The histogenesis of seminal vesicle CCA remains unclear. Some studies suggest that the histology of this tumor resembles CCA of the female genital tract, leading to speculation about Müllerian duct residual origin.^[9]^ This case, although presenting with histopathological features of CCA and negative CA125, did not fully conform to previous diagnostic criteria. However, the tumor’s histology completely matched that of ovarian CCA, and the tumor cells expressed specific markers CK7, PAX-8, NapsinA, and HNF-1β, consistent with the immunophenotype of female genital tract ovarian CCA. After consultations with urology and pathology departments of a higher-level hospital, it was unanimously agreed that this subtype shares homology with ovarian CCA of the female reproductive system.

While PSVA is a rare malignant tumor within the male reproductive system, its symptoms lack specificity. Patients often present with noticeable symptoms at a late tumor stage, leading to a poor prognosis, a short average survival time, and a 3-year survival rate of only 5%.^[12–14]^ The current treatment paradigm primarily involves radical surgical resection. Some scholars have advocated for a multidisciplinary team approach to diagnosis and treatment. While studies have indicated that a combination of radical surgery, radiotherapy, chemotherapy, and androgen deprivation therapy can be beneficial for patients with PSVA, the effectiveness of these treatments lacks validation through clinical follow-up.^[15]^ Therefore, further exploration of treatment modalities is necessary. In our case of primary seminal vesicle CCA with ovarian homology, we observed that patients generally have a better prognosis if the tumor is completely excised with negative margins. Incomplete resection or local metastasis should be supplemented with radiochemotherapy. In the future, gene sequencing, proteomics, and other technologies can be utilized to conduct in-depth comparative analyses of CCA originating from the seminal vesicle and ovary at the molecular level. This will facilitate the identification of more specific diagnostic biomarkers and potential therapeutic targets. For example, the expression levels and potential therapeutic value of targets like VEGF and PARP, already associated with ovarian CCA treatment, can be explored in seminal vesicle CCA. The discovery of novel diagnostic biomarkers will contribute to a more accurate differentiation of seminal vesicle CCA from other types of seminal vesicle tumors. This will provide clinicians with a more precise basis for prognostic assessment and treatment decision-making.

## Author contributions

**Conceptualization:** Yang Yuan, Yunlong Li, Song Xu, Tingting Gu.

**Data curation:** Yang Yuan, Yunlong Li, Shihao Zhang, Dingli Hu.

**Formal analysis:** Yang Yuan, Shihao Zhang, Dingli Hu.

**Funding acquisition:** Tingting Gu.

**Investigation:** Yang Yuan, Yunlong Li, Song Xu.

**Methodology:** Yang Yuan, Yunlong Li, Dingli Hu, Song Xu.

**Project administration:** Shihao Zhang.

**Resources:** Yang Yuan, Yunlong Li, Shihao Zhang, Song Xu, Tingting Gu.

**Software:** Shihao Zhang, Dingli Hu, Tingting Gu.

**Supervision:** Yunlong Li.

**Validation:** Tingting Gu.

**Writing – original draft:** Yang Yuan.

**Writing – review & editing:** Yang Yuan, Song Xu, Tingting Gu.
